# Exercise therapy, patient education, and patellar taping in the treatment of adolescents with patellofemoral pain: a prospective pilot study with 6 months follow-up

**DOI:** 10.1186/s40814-017-0227-7

**Published:** 2018-04-13

**Authors:** Michael S. Rathleff, Camilla R. Rathleff, Sinead Holden, Kristian Thorborg, Jens L. Olesen

**Affiliations:** 1 Research Unit for General Practice, Department of Clinical Medicine, Aalborg University, Aalborg, Denmark; 2 Department of Occupational Therapy and Physiotherapy, Department of Clinical Medicine, Aalborg University Hospital, Aalborg, Denmark; 3 Sports Orthopedic Research Center—Copenhagen (SORC-C), Department of Orthopedic Surgery, Copenhagen University Hospital, Hvidovre, Denmark; 4 Institute of Sports Medicine Copenhagen, Copenhagen University Hospital, Bispebjerg, Denmark

## Abstract

**Background:**

Patellofemoral pain (PFP) is the most common knee condition among adolescents, with a prevalence of 6–7% resulting in reduced function and quality of life. Exercise therapy is recommended for treating PFP, but has only been tested in older adolescents (15–19 years). This pilot study aimed to investigate the adherence to, and clinical effects of, exercise and patient education in young adolescents (12–16 years), with PFP.

**Methods:**

Twenty adolescents (16 females) with PFP were recruited from a population-based cohort to undergo a 3-month multimodal intervention. This comprised of a 30-min patient education and group-based exercise therapy. Exercises included supervised lower extremity strength exercises three times per week, in addition to similar home-based strength exercises. Outcomes included a 7-point global rating of change scale (ranging from “completely recovered” to “worse than ever”), the Knee injury and Osteoarthritis Outcome Score (KOOS), physical activity scale (PAS), weekly sports participation and health-related quality of life measured by European Quality of Life 5 dimensions Youth (EQ-5DY) and isometric knee and hip muscle strength. Pain was measured on a visual analogue scale (VAS), and satisfaction treatment was measured on a five-point Likert scale ranging from “highly satisfied” to “not satisfied at all”. These were collected at 3- and 6-month follow-ups. Adherence to supervised exercise was measured as session attendance, and adolescent self-reported adherence to home-based exercises.

**Results:**

Adherence to the exercise therapy was poor, with adolescents participating in a median of 16 (IQR 5.5–25) out of 39 possible supervised training session. Five out of 18 adolescents had a successful outcome after both 3 and 6 months. There were no relevant changes in isometric muscle strength.

**Conclusion:**

This was the first study to investigate adherence to, and clinical effects of, exercise therapy and patient education in young adolescents with patellofemoral pain. Adherence to the exercise therapy was low with little to no clinical effects making a full clinical trial impractical. Future studies need to explore how an intervention can be successfully tailored to young adolescents with patellofemoral pain to obtain good adherence while improving pain and function.

## Background

There is an 8-fold increase in the number of general practice consultations for knee problems between the ages of 5–9 and 10–19 [1, 2]. Patellofemoral pain (PFP) is the most common knee complaint, affecting 6–7% of adolescents [3, 4]. PFP is characterised by diffuse anterior knee pain, which is provoked by squatting, prolonged sitting, and stairclimbing [5]. Pain can often be long-standing, which will impact function and health-related quality of life [5].

Exercise therapy is one of the foundations for treating PFP, being recommended by both a Cochrane review and expert consensus [6, 7]. A previous cluster randomised trial in older adolescents (15–19 years of age) with PFP, demonstrated that the addition of exercise therapy to patient education improved recovery in the short- (3 months), and long-term (24 months) [8]. However, the recovery rate was lower than what has previously been observed among adults, with only one-third being recovered at follow-up [9].

Adolescents with PFP in this study by Rathleff et al. [8] already had a symptom duration of more than 3 years, with only 5% reporting a symptom duration less than 6 months. This symptom duration is longer than in previous trials on adults. van Linschoten et al. [8] reported that the majority (nearly 68%) of patients in their trial had symptoms for 2–6 months, while the median symptom duration reported by Collins et al. [9] was 28 months, with only 25% having symptoms for less than 12 months. This is important, because previous studies show that longer symptom duration is associated with a poorer outcome after treatment [10]. The differences in symptom duration between adolescents and adults may explain the lower overall effect observed in adolescents by Rathleff et al. [9]. If true, then perhaps the efficacy of exercise therapy in adolescents can be increased by targeting younger adolescents with a shorter symptom duration. No studies have investigated the delivery of supervised exercise to younger adolescents with PFP.

Therefore, the purpose of this pilot study was to investigate the adherence to, and effect of an exercise therapy intervention, on a self-reported Global Rating of Change (GROC), knee function (KOOS) and muscle strength in young adolescents with PFP (12–16 years of age). Specifically, the aim was to explore adherence to exercise therapy and to use the patient reported outcomes to inform a sample size calculation for a definitive trial.

## Methods

### Study design

This study was designed as a cohort study, including 20 adolescents with PFP. Participants were recruited from a population-based cohort (Adolescent Pain in Aalborg 2011, the APA2011-cohort) [11]. The reporting of the study complies with the STROBE reporting guideline and the TIDieR checklist for reporting of interventions [12, 13].

### Recruitment

In September 2011, eight lower secondary schools in Aalborg were invited to answer an online questionnaire and to be part of the Adolescent Pain in Aalborg 2001 (APA2011)-cohort. A total of 768 students aged 12–15 years answered the online questionnaire, with 215 (28%) reporting knee pain. Those reporting knee pain were contacted in September 2012. They were offered a clinical examination by an experienced rheumatologist if they still had knee pain and fulfilled the following criteria: pain for more than 6 weeks; insidious onset of knee pain felt anteriorly around the patella or diffusely around the knee; no treatment within the previous 12 months, and no previous knee surgery.

### Inclusion and exclusion criteria

Eligibility criteria for inclusion were applied by the rheumatologist at the clinical exam, and were in line with a previous clinical trial [2] as follows:

1. Insidious onset of anterior knee or retropatellar pain of greater than 6 weeks’ duration;
2. Pain provoked by at least two of the following situations: prolonged sitting or kneeling, squatting, running, hopping, or stair walking;
3. Tenderness on palpation of the patella, or pain with stepping down or double leg squatting;
4. Worst pain during the previous week of more than 30 mm on a 100 mm visual analogue scale (VAS).

Exclusion criteria were the following:

1. Concomitant injury or pain from the hip, lumbar spine, or other knee structures;
2. Previous knee surgery;
3. Patellofemoral instability;
4. Knee joint effusion;
5. Use of physiotherapy for treating knee pain within the previous year;
6. Weekly use of anti-inflammatory drugs.

### Intervention

One physiotherapist delivered the multimodal intervention (patient education, exercise therapy and patella taping, Table 1) to all participants. She was previously involved in administering this intervention to older adolescents (15–19 years of age) with PFP [8]. The intervention was an exact replication of the intervention delivered in that RCT [8, 14].

**Table 1:** TIDieR checklist for reporting of interventions

Intervention name	Why	What (materials and procedure)	Who provided	How?	Where did the intervention take place	When and how much?	Tailoring	Modification	How well? (fidelity and adherence)
Exercise therapy and patient education	This multimodal program has never been tested in young adolescents with PFP, only among 15–19 year olds with PFP.	One physiotherapist delivered the patient education, exercise therapy, and instructions on patellar taping. The exercise therapy was based on previous trials and consisted of a combination of supervised group training sessions and unsupervised home-based exercises.	Physiotherapist	Face to face	At the hospital	The unsupervised home exercises consisted of approximately 15 min of quadriceps and hip muscle retraining and stretching and were performed every day except for the days of supervised sessions. The supervised exercises were offered three times per week at the hospital for 13 weeks. Full description of intervention can be seen in this open access publication [3]	To progressively match the exercise level to the performance level and pain levels of each participant, all exercises were available in multiple levels of difficulty.	All adolescents started with exercises at level 1 and progressed from there. The progression followed previously described rules. (1) Good quality of movement determined by the physiotherapist. ‘Good quality’ is defined as able to control hip, knee, and foot alignment during exercises with both extra-slow and slightly faster than normal movement. (2) Ability to perform the actual number of repetitions as defined in the training protocol. (3) No self-reported increase in usual pain after the training session or the next morning.	Adherence to the supervised sessions was recorded as attendance. The adolescents participated in a median of 16 (IQR 5.5–25) supervised training session during the 13 weeks

### Patient education

Patient education lasted for approximately 30 min and was standardised and covered the following topics: (1) why does it hurt, (2) pain management, (3) how to modify physical activity, (4) how to return slowly to sports, (5) how to cope with knee pain, (6) information on optimal knee alignment during sit-to-stand, standing, walking, stair walking and bicycling, and, (7) questions from the adolescent or the parents. Adolescents received information both face to face and in an 8-page leaflet.

### Exercise therapy

Exercise therapy was delivered and supervised by one physiotherapist who had previous experience in treating PFP, and more than 3 years of practical experience with adolescents and group-based exercises. Group-based exercise sessions were offered at the local hospital three times per week. All exercises were available in three to four different levels to allow for tailoring to each adolescent’s performance and to enable progression in load and difficulty (i.e. balance and control) of the exercises. Adolescents started at level one and progressed from there. Progression was made on three general rules:

1. Good quality of movement (determined by the physiotherapist). This was defined as being able to control hip, knee, and foot alignment during exercises with both extra-slow and slightly faster than normal movement.
2. Ability to perform the complete number of repetitions defined in the training protocol.
3. No increase in usual pain after the training session or the next morning.

Further, the physiotherapist could adjust the external weight, repetitions, and sets based on pain. Generally, adolescents started with a load of 15 repetition maximum (RM), progressing to three sets of 8–10 RM. Adolescents were instructed to inform the physiotherapist if they felt their pain exceeded 3 cm on a Numeric Rating Scale (NRS), and the load was adjusted accordingly.

To account for variation in the time the school lessons end and improve adherence, students were offered the opportunity to attend the supervised group training session at 15:00 or 16:00. Group-based training sessions were available three times per week (Mondays, Wednesdays, and Fridays) for 3 months (corresponding to 13 weeks or a total of 39 training sessions).

### Patellar taping

Patella taping was used if patients achieved a minimum of 50% reduction in pain (measured by VAS) during a two-leg squat immediately after application of tape. The patellar taping was based on the McConnell approach as it may reduce pain during exercise [12]. Non-rigid, hypoallergenic tape (Curafix H, Lohmann and Rauscher, Neuweid, Germany) was used to reduce skin irritation, while rigid zinc-oxide tape (Leuko P, BSN Medical, Hamburg, Germany) was applied to correct the position of the patella. Taping corrections were applied in a predetermined order of anterior tilt, medial tilt, glide, and fat pad unloading until the participant**’**s pain was reduced by at least 50% [15]. If the taping did not reduce pain, participants were not instructed to use it. If the taping reduced pain, adolescents were taught to independently apply the taping corrections and instructed to reapply daily, and to wear it during waking hours for the duration of the intervention period.

### Home exercises

In addition to the supervised group training, adolescents were instructed to perform home exercises four times per week. They were also instructed to perform home exercise if there were any days where they missed the supervised exercises. Home-based exercises consisted of quadriceps and hip muscle exercises, and stretching [8]. These were also included during the supervised exercise therapy to ensure adolescents were well instructed in the home exercises. Adolescents were advised to continue to perform home exercises after the 3 months of group-based exercises finished. No specific time-period was given.

### Adherence

Parents were invited to participate in all aspects of the study, with a hope to optimise adherence to the intervention and increase retention. Communication was done through telephone or email. The day before appointments, adolescents were sent an SMS reminder. Participants were asked to send an SMS to the physiotherapist, if they could not participate in the group training sessions. If students did not show up for training twice in a row, without cancelling through SMS, they were telephoned by the physiotherapist who asked them in a friendly manner when they would return.

Adherence to supervised exercise therapy was recorded as attendance at the supervised classes. The physiotherapist recorded participation in the group-based exercises at each session. We defined good adherence as participation in at least 80% of the supervised group training sessions, as was previously used in adolescents with PFP [8]. Poor adherence was defined as participation in less than 40% of the group training sessions [8]. Adherence to the home-based exercises were based on self-report data from a training log from the adolescents.

### Concurrent interventions

Adolescents were asked to refrain from other interventions during the intervention period, starting 72 h before participation in the study. Pre-existing foot orthoses were allowed, but they were not allowed to change or modify their current orthoses during the study period. Current or prior analgesic use for the current knee pain was registered during baseline testing and all follow-ups. However, we note the small sample size, and these results should be interpreted with caution.

### Outcome measurements

Adolescents filled in self-report questionnaires at baseline, 3, and 6 months. The questionnaires were trialled successfully among a small group of 3–5 adolescents before we used them in the current study.

A priori, we defined that our primary measures of interest were adherence to the intervention, and the number of adolescents with a successful outcome. We deemed these two measures relevant to inform feasibility and sample size requirements for a potential future trial. Successful outcome was measured on a 7-point Likert scale ranging from “completely recovered” to “worse than ever”. Identical to van Linschoten et al. patients were categorised as having a successful outcome if they rated themselves as “fully recovered” or “strongly recovered”, whereas those who rated themselves as “slightly recovered” to “worse than ever” were categorised as not having a successful outcome [16]. Other outcomes were the Knee injury and Osteoarthritis Outcome Score (KOOS) [17], physical activity scale (PAS) [18], and weekly sports participation (number of times per week). Health-related quality of life was measured by the European Quality of Life 5 dimensions Youth (EQ-5DY) [19]. Worst pain during the past week and pain during usual activity were measured on a visual analogue scale (VAS). Treatment was measured on a 5-point Likert scale ranging from “highly satisfied” to “not satisfied at all”. Adolescents were allowed ask their parents if they were unsure about the meaning of the questions.

In addition to the self-report outcomes, isometric strength was measured before and after the 3-month intervention. The testing setup included a portable dynamometer and an examination table as described in our previous described methods [20]. Muscle strength was tested with the Mecmesin AFG2500 dynamometer, which was bolted to the wall to ensure fixation. All strength tests were tested isometrically. Six movement directions around the knee and hip were tested as follows: knee flexion and extension; hip abduction and adduction; hip internal and external rotation. The dynamometer strap was positioned 5 cm proximal to the medial malleolus, perpendicular to the anterior or posterior aspect of the tibia [20]. Knee extension strength was tested with the knee in 60° flexion, while flexion strength was tested during 90° of knee flexion. Hip abduction and adduction were tested with the participant lying supine on the examination table. The strap was positioned 5 cm proximal to the medial malleolus, perpendicular to the medial or lateral aspect of the tibia. The leg was placed in 0° flexion and abduction. Hip internal and external rotation strength were tested with the participant sitting on one side of the examination table, with the hip and knee flexed at 90°. The reliability of these tests was high, with ICC values for all six movement directions were above 0.92 [20].

### Sample size

As this was a pilot study, no formal sample size calculation was undertaken. Twenty adolescents were included to inform a proper sample size calculation for a future trial from the data on the adherence to the exercise intervention, and the results from the 7-point Likert scale (ranging from “highly satisfied” to “not satisfied at all”).

### Statistical analysis

All data were visually inspected for normality using a Q-Q plot. Mean values ± SD are reported if data were normally distributed. If data were non-normally distributed, they were presented as median and interquartile range (IQR). Paired samples *t* tests were used to test the changes in patient reported outcomes and isometric strength between matched pairs. Mann-Whitney *U* tests were used to compare adherence to home-based exercises between adolescents with good and poor adherence to supervised exercises. *P* < 0.05 was considered statistically significant. All calculations were performed using Stata version 11 (StataCorp, College Station, TX, USA).

## Results

### Participants

Twenty adolescents between 12 and 16 years of age were included (Table 2). Eighteen of these 20 adolescents participated in the follow-up after 3 months and 18 of the 20 participated in the follow-up at 6 months. Ten adolescents responded to patellar taping and were advised to continue using it throughout the 13 weeks.

**Table 2:** Participant characteristics

	Adolescents with PFP *N* = 20
Age [years]	14.6 (± 1.1)
Height [cm]	167.0 (± 10.0)
Weight [kg]	55.2 (± 9.0)
Gender (number of females)	16
BMI [kg/m^2^]	19.5 (18.2–20.7)**
Pain duration [months]	28.5 (24–36)**
Sports participation [times per week]	4 (3–4.5)**
Regular use of pain killers (number of adolescents)	11

The variables are presented as mean and standard deviation or median and inter-quartile range. *BMI* body mass index

**Presented as median and interquartile range

### Adherence

**Fig. 1:**
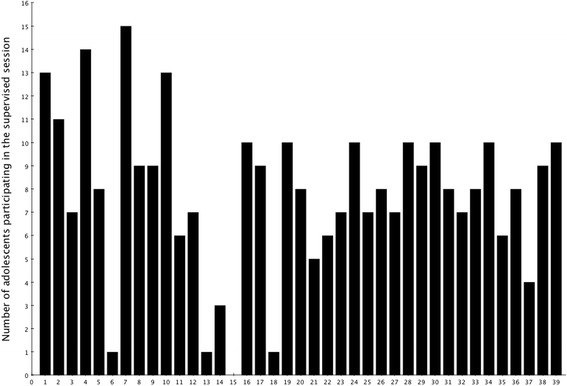
Adherence during the 3 months. In total, adolescents were offered to attend 39 supervised training sessions

Adolescents participated in a median of 16 (IQR 5.5–25) supervised training session during the 13 weeks (Fig. 1). None participated in more than 80% of the 39 supervised training sessions, with 40% participating in less than 40% (therefore, being characterised as having poor adherence). At least one parent of each child took part in the education session, but no parents took part in the supervised exercise sessions despite being invited.

During the first 3 months, adolescents reported performing a median of 2 (IQR 2–3) home training sessions per week, with a median of 26 home-based training sessions over the course of the 13 weeks (corresponding to 50% of the prescribed home exercise dosage). Adolescents with poor adherence to supervised training reported a slightly higher adherence to home-based exercises compared to compliant adolescents (3 (IQR 3–4) vs 2 (IQR 2–2) times per week; *p* = 0.002), corresponding to 75% of the prescribed home exercise dosage. Six adolescents reported still performing their home exercises at 6 month follow-up (median of 2.5 times per week (IQR 2–3)), while the rest reported that they had stopped.

### Recovery, satisfaction with the treatment and co-interventions

Five adolescents were categorised as having a successful outcome after 3 months. Similarly, 5/18 adolescents had a successful outcome after 6 months (only three of these had a successful outcome at both time-points). Eleven out of 18 reported being highly satisfied or very satisfied with the results of the treatment after 3 months. Nine out of 18 were either highly satisfied or very satisfied with the results of the treatment after 6 months. There were only small improvements in self-reported outcomes (Table 3).

**Table 3:** Patient-reported outcome measures and physical activity level

	Baseline (± SD)	3-month follow-up (± SD)	6-month follow-up (± SD)	Mean change from baseline to 3 months (95% CI)	Mean change from 3 to 6 months (95% CI)	Mean change from baseline to 6 months (95% CI)
KOOS pain	71 (± 13)	70 (± 16)	76 (± 14)	1 (− 8; 9)	6 (− 1; 10)	5 (− 2; 11)
KOOS symptom	76 (± 11)	77 (± 12)	81 (± 11)	0 (− 7; 6)	4 (− 1; 7)	5 (− 2; 11)
KOOS activities of daily living	79 (± 13)	82 (± 14)	87 (± 10)	2 (− 5; 9)	5 (0; 9)	7 (0; 13)
KOOS sport and recreation	58 (± 19)	62 (± 21)	68 (± 21)	4 (− 7; 14)	6 (− 4;16)	9 (1; 18)
KOOS quality of life	54 (± 12)	59 (± 21)	59 (± 21)	4 (− 4;11)	0 (− 10;10)	4 (− 4; 12)
Physical activity level (physical activity scale (METs))	45.0 (± 8.3)	49.4 (± 10.7)	55.4 (± 13.0)	4.8 (− 1.8; 11.3)	6.2 (− 1.1;13.6)	10 (2; 19)
Health-related quality of life (EQ5D index**)	0.75 (0.72–0.78)	0.78 (0.72–0.82)	0.82 (0.72;0.84)			
Self-related health (EQ-VAS)	76 (± 22)	78 (± 15)	78 (± 17)	− 1 (− 8; 9)	0 (− 8; 8)	1 (− 8; 6)
Worst pain last week (VAS* worst)	62 (± 19)	51 (± 27)	48 (± 27)	− 10 (− 22; 3)	− 3 (− 14; 8)	− 13 (− 27; 1)
Pain during activity (VAS* activity)	56 (± 19)	37 (± 26)	37 (± 22)	− 18 (− 30; 6)	− 1 (− 7; 5)	− 19 (− 30; − 8)

*VAS visual analogue scale

**Reported as median and interquartile range

### Isometric muscle strength

There were no significant changes in knee strength from before to after the 3-month intervention. While there were some small significant changes in hip strength, these did not exceed previously established limits of agreement for hip strength (Table 4).

**Table 4:** Isometric muscle strength at baseline and 3-month follow-up

	Baseline (±SD)	Follow-up at 3 months (±SD)	Difference (95% CI)	*p* value
Knee extension (%BW)	0.82 (± 0.21)	0.84 (± 0.23)	0.01 (− 0.04; 0.06)	0.59
Knee flexion (%BW)	0.33 (± 0.07)	0.33 (± 0.07)	0.01(− 0.02; 0.03)	0.63
Hip abduction (%BW)	0.26 (± 0.05)	0.24 (± 0.05)	− 0.02 (− 0.03; 0.00)	0.03
Hip adduction (%BW)	0.27 (± 0.07)	0.24 (± 0.05)	− 0.03 (− 0.05; − 0.01)	0.01
Hip external rotation (%BW)	0.21 (± 0.04)	0.23 (± 0.05)	0.01 (0.00; 0.02)	0.05
Hip internal rotation (%BW)	0.32 (± 0.06)	0.34 (± 0.07)	0.02 (0.00; 0.04)	0.09

### Concurrent interventions

One adolescent reported “acupuncture” and one adolescent visited a physiotherapist outside the study, during the 13 weeks of intervention. Two adolescents reported visiting a physiotherapist between the 3 and 6 months follow-up. Nine adolescents used pain-killers at 3 months follow-up, and 11 used pain-killers at 6 months follow-up.

## Discussion

Our primary measures of interest were adherence to, and the clinical effects of, exercise therapy and patient education in young adolescents with PFP. Due to the shorter symptom duration compared to our previous trial in older adolescents [2], we expected the intervention would have a stronger effect in this young adolescent population. Contrary to this, the intervention demonstrated no clear effect, with low adherence to the intervention, and little involvement from the parents.

As with older adolescents [8, 21], adherence to supervised exercise therapy was low, with 40% attending less than 40% of all supervised training sessions, and none being classified as having good adherence (partaking in > 80% of training sessions). Over the 13-week intervention, participants attended a median of 18 supervised training sessions (compared to 8.5 in the previous RCT in older adolescents [8]), and 26 home-based training sessions. Despite similar (if not slightly higher) adherence, the proportion of successful outcomes at 6 months seemed slightly lower in this pilot study. Considering the poor adherence and clinical effects, this suggests that supervised exercises offered three times per week for 3 months may not be optimal for this age group. The adolescents reported participating in a higher number of home-based unsupervised training session compared to their participation in supervised training sessions. This suggests that home-based intervention may result in better adherence in this young population. This would potentially be preferable due to its less time intensive nature, lower cost, and ease of implementation. Recent research suggests the environment contributes to treatment response [22]. As such, matching patients’ preferences to treatment rooms may be a way to improve outcome and adherence [22]. Future studies need to explore the reasons for poor adherence, and if/how interventions may be better tailored to adolescents’ preferences to improve adherence.

Unfortunately, the intervention did not improve hip or knee strength, despite its previous efficacy in older adolescents with PFP [23]. This could be due to the previously mentioned problems with compliance. On the other hand, it has been shown that younger adolescents with PFP do not yet display similar strength deficits [20] as older adolescents (age 15–19) [20] and adults with PFP [24]. One study even demonstrated that *increased* hip strength was a risk factor for PFP in adolescent females [25]. An important consideration is that, PFP is often associated with high sports participation, with one in four adolescent female athletes suffering from it [26]. The adolescents in this study still participated in sport on average, four times per week despite long-standing knee pain, and may not yet have developed strength deficits due to absence from their sport. If these young adolescents had not yet developed strength deficits, this may explain why strength training was not as effective in this population. As previous research indicates that individuals with the largest strength deficits benefit most from exercise therapy [27], there is no strong rationale for focussing solely on exercise therapy and strength improvements in this population.

Participants in this cohort had an average pain duration of 28 months, despite their young age. As pain duration is one of the most consistent predictors of poor outcome [10], there is a need to identify better treatment strategies to intervene in young adolescents with early onset of PFP. It has previously been highlighted that there is a difference in the responsiveness of adolescents and adults with PFP to exercise therapy, despite similar exercise adherence [9]. Current exercise-focused treatments are only effective for 30–40% of adolescents (15–19 years of age) with PFP [8]. One of the plausible reasons for lack of effectiveness is that exercise therapy, on its own, does not help the adolescents modify and control their sports participation. Our previous research demonstrates that adolescents with PFP continue to participate in high-level sports despite long-standing and intense knee pain. One solution may be to investigate activity modification and load management to teach the adolescent how to modify and control their sports participation based on their knee symptoms.

Currently, there is a lack of research examining management of PFP [1] in adolescents, despite the fact that it is the most common and persistent knee condition seen in this population [8, 28]. One previous RCT, using the same comprehensive intervention as the current investigation, demonstrated that less than 40% had a successful outcome after 1 year. Taking the poor adherence in this pilot study together with this underscores the need for better tailored management strategies for this young population. Future studies need to explore interventions that can obtain good adherence, engage parents to reinforce adherence, and include activity modification and load management. We believe these steps will be vital to address in order to develop optimal intervention strategies specifically tailored to the lives and needs of adolescents.

### Limitations

This was a pilot study on a small cohort of young adolescents with PFP. There was no control group preventing us from understanding this intervention compared to other treatment strategies. The patient-reported outcomes were supposed to be used to inform a sample size calculation for a full clinical trial. However, adherence to the intervention was poor, and there were only small clinical effects of the intervention, making a full clinical trial impractical. Additionally, as we did not measure physical activity levels throughout the study with objective methods such as accelerometer, it is unknown how physical activity changed during the study or if this is associated with successful outcomes.

## Conclusion

This was the first study to investigate adherence to, and clinical effects of, patient education and exercise therapy in young adolescents with PFP. Adherence was low, as was the clinical effect from this approach. This suggests that supervised exercises three times per week after school hours are not optimal in this population. It therefore seems important to develop new and better strategies for adolescents with PFP. One approach could include load management and more patient empowerment on how the adolescents can manage their pain and sports participation at home to help them return safely to their sports.
